# Deubiquitinase MYSM1: An Important Tissue Development and Function Regulator

**DOI:** 10.3390/ijms252313051

**Published:** 2024-12-04

**Authors:** Qiaozhen Qin, Huaqiang Ruan, Heyang Zhang, Zhenhua Xu, Wenting Pan, Xinlong Yan, Xiaoxia Jiang

**Affiliations:** 1 Beijing International Science and Technology Cooperation Base for Antiviral Drugs, Beijing Key Laboratory of Environmental and Viral Oncology, College of Chemistry and Life Science, Beijing University of Technology, Beijing 100124, China; qqzhen32477@163.com (Q.Q.); pwting163@163.com (W.P.); 2 Beijing Institute of Basic Medical Sciences, Beijing 100850, China; hq.ruan@foxmail.com (H.R.); 18638863853@163.com (H.Z.); doctorzhenhua@foxmail.com (Z.X.)

**Keywords:** deubiquitinase, MYSM1, stem cells, immune cells, cancer, aging, depression

## Abstract

MYSM1, a deubiquitinating enzyme, plays a pivotal role in diverse biological processes. Both MYSM1 knockout mice and patients with Mysm1 gene mutations exhibit developmental abnormalities across multiple tissues and organs. Serving as a crucial regulator, MYSM1 influences stem cell function, immune responses, and the pathogenesis of diverse diseases. This review comprehensively details MYSM1’s deubiquitinating activities in both the nucleus and cytoplasmic compartments, its effects on stem cell proliferation, differentiation, and immune cell function, and its involvement in cancer, aging, and depression. The high sequence homology between murine and human MYSM1, along with similar phenotypes observed in Mysm1-deficient models, provides valuable insights into the etiology of human Mysm1-deficiency syndromes. This review aims to offer a foundation for future comprehensive research on MYSM1.

## 1. Introduction

Myb-like, SWIRM, and MPN domain 1 (MYSM1) is a deubiquitinating enzyme characterized by the presence of SANT, SWIRM, and MPN domains [1]. It plays critical roles in regulating the development and function of various tissues and organs. MYSM1 exhibits high expression in the hematopoietic system, particularly in hematopoietic stem cells (HSCs) [2] and their derivatives, such as B cells [3], T cells [4], and natural killer (NK) cells [5]. In addition, MYSM1 is prominently expressed in the central nervous system, especially in astrocytes [6]. MYSM1 regulates the expression of key transcription factors like Id4, thereby influencing the proliferation and differentiation of neural stem cells (NSCs) [7]. Additionally, MYSM1 expression has been identified in various tumor cells, including castration-resistant prostate cancer (CRPC) cells [8], colorectal cancer (CRC) cells [9], melanoma cells [10], ERα-positive breast cancer (BC) cells [11], and triple-negative breast cancer (TNBC) cells [12].

The MYSM1 sequence is highly conserved across primates, mice, and humans, and the phenotypes resulting from Mysm1 deficiency are remarkably similar across these species. For instance, both Mysm1 knockout mice and patients with Mysm1 gene mutations exhibit a severe depletion of B cells and NK cells, accompanied by moderate reductions in T cells, myeloid cells, and erythroid cells [13]. Additionally, Mysm1 knockout mice also display progressive age-related bone loss [14], which parallels the abnormal bone development in patients with Mysm1 mutations. Moreover, recent studies have shown that both Mysm1-deficient mice and patients with Mysm1 gene mutations exhibit abnormal brain development [7].

In addition to its role in development, MYSM1 is closely associated with tumorigenesis across various cancer types. Studies have shown that MYSM1 expression exhibits distinct pattern in different cancers: it is notably reduced in certain colorectal cancer tissues compared to in normal tissues [9], while showing significant elevation in some melanoma tissues relative to the surrounding tissues [10]. Mysm1 deficiency triggers p53 activation in hematopoietic cells, indicating its potential as a therapeutic target in hematological malignancies with active p53 [4]. In colorectal cancer, a lower MYSM1 expression correlates with a better prognosis, as it epigenetically upregulates the miR-200 family and CDH1, subsequently suppressing the PI3K/AKT pathway [9]. Furthermore, MYSM1 downregulation in CRPC promotes cell proliferation and helps tumor cells, evading senescence under androgen ablation [8]. Notably, in TNBC, the restoration of MYSM1 increases cisplatin-induced apoptosis, highlighting its various roles in cancer progression and treatment [12].

Recent studies have demonstrated that MYSM1 expression is substantially increased in the brains of both depression patients and mice displaying depressive-like behaviors [6]. Moreover, therapeutic interventions specifically targeting MYSM1 expression in the brain have shown remarkable efficacy in alleviating depressive-like symptoms in mouse models [6,15,16].

This review provides a comprehensive overview of MYSM1’s deubiquitination functions in regulating stem cell and immune cell activities, and its involvement in cancer, aging, and depression, providing valuable insights for future comprehensive research in the MYSM1 field.

## 2. MYSM1’s Deubiquitination Functions

### 2.1. MYSM1’s Role in Monoubiquitination Removal

**Figure 1:**
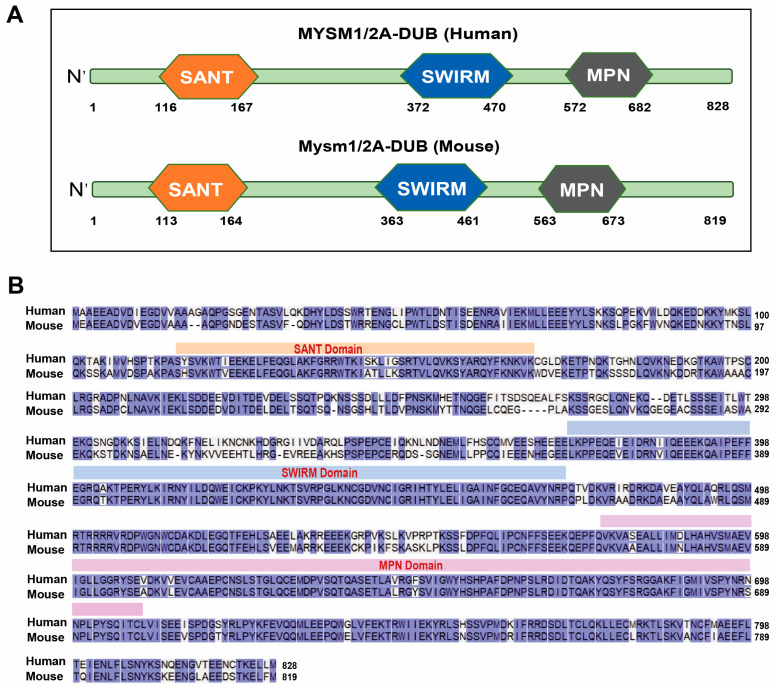
(**A**) Domain structure of human and mouse MYSM1 proteins, which comprise SANT, SWIRM, and MPN domains. (**B**) Amino acid sequence of human and mouse MYSM1 proteins.

MYSM1 is a histone-deubiquitinating enzyme comprising SANT, SWIRM, and MPN domains, which displays up to 87% homology between human and mouse MYSM1 proteins (Figure 1). The consistent phenotypes observed in Mysm1 deficiency across different species suggests that studying MYSM1 in mouse models could provide substantial insights into the etiology of human Mysm1 deficiency syndromes. Structurally, the SANT domain of MYSM1 resembles the DNA-binding domain of the transcription factor cMYB [17]. While this domain has demonstrated a DNA-binding capacity [18], whether this binding is sequence-specific is still unclear. Unlike its counterparts found in human ADA2α and SMARC2, the SWIRM domain of MYSM1 lacks DNA-binding activity [1]. However, this domain is hypothesized to modulate the functions of both SANT and MPN domains through interactions with other proteins, particularly with histones, thus participating in gene expression regulation. The MPN metalloprotease domain serves as the catalytic core of MYSM1, characterized by its association with Zn^2+^ and deubiquitinating enzyme (DUB) activity [1,19,20].

Histone H2A monoubiquitination at lysine 119 (H2AK119ub) was initially identified as a substrate of MYSM1 [21]. Acting as a repressive epigenetic mark, H2AK119ub is predominantly deposited on chromatin by the polycomb repression complex 1 (PRC1), which facilitates long-term gene silencing during cellular differentiation and lineage specification [22,23,24]. In contrast, histone H2BK120ub serves as an epigenetic mark associated with transcriptionally active genes [25,26]. MYSM1 primarily activates gene expression through its deubiquitinating enzyme activity, specifically targeting histone H2AK119ub rather than H2BK120ub [21]. Consequently, the catalytic removal of H2AK119ub by MYSM1 thus reinforces its predominant role in gene activation.

Although MYSM1 can catalyze the deubiquitination of H2AK119ub, several other specific DUBs, including BAP1 and USP16, also show specificity for H2AK119ub. Additionally, enzymes such as USP3, USP12, USP22, and USP44 can deubiquitinate both H2AK119ub and H2BK120ub [27]. However, the mechanisms by which MYSM1 cooperates with other DUBs to regulate histone H2A ubiquitination and subsequent gene expression patterns across various mammalian cell types remain unclear.

### 2.2. MYSM1’s Role in Polyubiquitination Removal

**Figure 2:**
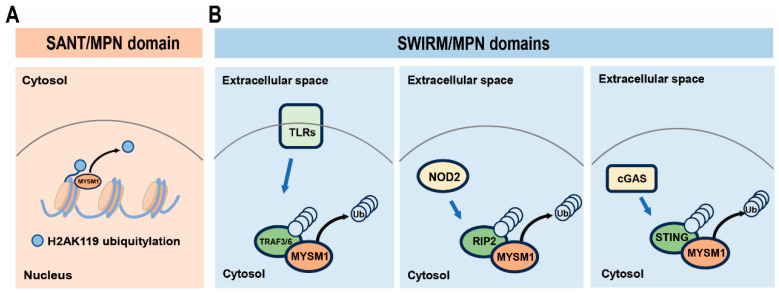
Overview of MYSM1 protein functions in the nucleus and cytosl. (**A**) MYSM1 functions as a deubiquitinase that specifically cleaves monoubiquitin from H2AK119ub in the nucleus. (**B**) MYSM1 mediates the deubiquitination of key signaling molecules, including TRAF3, TRAF6, RIP2, and STING, in pattern recognition receptor pathways (TLR, NOD2, and cGAS), thus repressing the innate immune and inflammatory responses.

In addition to removing monoubiquitination at H2AK119, MYSM1 also effectively removes polyubiquitin chains (Figure 2A). In vitro assessments of MYSM1’s activity on various polyubiquitin chain configurations revealed its ability to cleave M1, K6, and K27 chains, while exhibiting reduced activity on K11, K29, K33, and K48 chains [26,28]. In macrophages, MYSM1 also dissociates K63-linked ubiquitin chains from proteins such as TRAFs, RIP2, and STING, which are involved in signaling cascades associated with Toll-like (TLR), NOD2, and cGAS receptors, thereby dampening inflammatory responses [18,28] (Figure 2B). Thus, MYSM1 functions as a versatile protein with a broad spectrum of deubiquitination substrates. This diversity suggests that our current understanding of MYSM1’s substrates is incomplete, and future advances in scientific research and technology are expected to uncover additional regulatory targets of MYSM1.

## 3. MYSM1’s Role in Regulating Stem Cell Functions

### 3.1. MYSM1’s Role in Hematopoietic Stem Cell Proliferation and Differentiation

**Figure 3:**
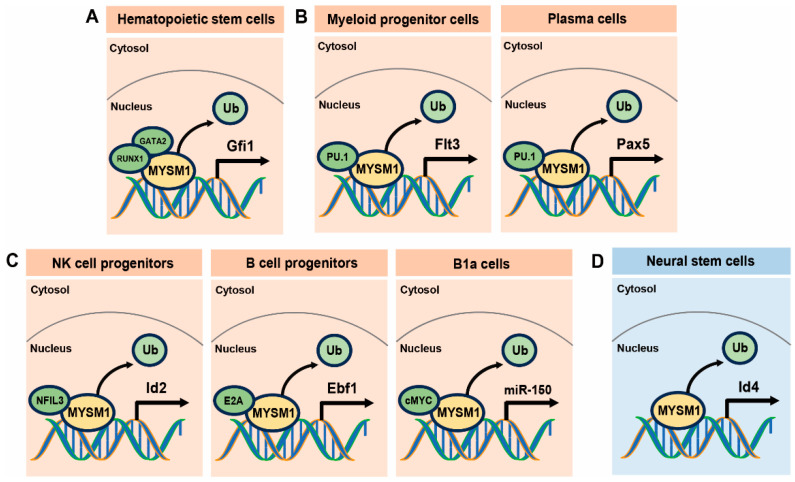
Schematic model illustrating MYSM1’s function in regulating hematopoietic and neural stem cells. (**A**) MYSM1 enhances Gfi1 expression in hematopoietic stem and progenitor cells. (**B**) MYSM1 promotes Flt3 expression in myeloid progenitor cells and enhances Pax5 expression in plasma cells. (**C**) MYSM1 enhances multiple targets: Id2 expression in NK cell progenitors, Ebf1 expression in B cell progenitors, and miR-150 expression in B1a cells. (**D**) MYSM1 influences NSC proliferation and differentiation by modulating Id4 transcription. In Mysm1-deficient NSCs, hyperproliferation occurs alongside enhanced neurogenesis and compromised astrogliogenesis.

Following the discovery of hematopoietic dysfunction in Mysm1-deficient mouse models, subsequent research has predominantly focused on the transcriptional regulation of MYSM1 in hematopoietic cells [29]. These studies have elucidated the deubiquitination role of MYSM1 in regulating a series of genes essential for normal stem cell differentiation and hematopoietic lineage specification [29]. These MYSM1-regulated genes include Ebf1 in B-cell progenitors [30], Pax5 in naïve B cells [3], miR150 in B1a cells [31,32], Id2 in NK cell progenitors [5], Flt3 in dendritic cell precursors [33], and Gfi1 in HSCs and hematopoietic progenitor cells (HPCs) [2]. At the molecular level, MYSM1 promotes the transcription of Gfi1 by regulating histone modifications and recruiting key transcription factors such as Gata2 and Runx1, thus elucidating the mechanism of MYSM1 in HSC homeostasis and potential epigenetic regulatory pathways (Figure 3A) [2]. MYSM1 also regulates the development of dendritic cells (DCs) from common myeloid progenitors (CMPs) by modulating histone modifications on the Flt3 gene promoter, thereby promoting Flt3 transcription. This epigenetic control is essential for proper hematopoiesis and immune cell development (Figure 3B) [33]. While mice deficient in MYSM1 show severe defects in B cell development, they exhibit an enhanced antibody response to both T cell-dependent and independent antigens. Researchers have previously revealed that MYSM1 intrinsically represses plasma cell differentiation and antibody production by acting as a transcriptional activator of Pax5, which suppresses plasma cell differentiation and antibody production by facilitating key transcriptional factor recruitment and coordinating histone modifications at the Pax5 loci (Figure 3B) [3]. In mice lacking MYSM1, NK cell development is severely impaired, highlighting MYSM1’s essential role in NK cell maturation without affecting the NK lineage specification and commitment. MYSM1 regulates Id2 expression by interacting with the nuclear factor IL-3 (NFIL3), thereby promoting the development of NK cells and demonstrating its significant epigenetic regulatory role (Figure 3C) [5]. MYSM1 also plays an essential role in early B cell development, as its deficiency in mice results in early B cell commitment blockage and an impaired B cell progenitor expression of Ebf1 and other B lymphoid genes (Figure 3C) [30]. Additionally, MYSM1 is crucial for the proliferation of B1a cells, and its deficiency leads to increased B1a cell proliferation in mice. MYSM1 promotes the transcription of miR-150 by recruiting c-Myc, which reduces Flt3 expression in B1a cells. The higher proportion of Flt3^+^ B1 cells in systemic lupus erythematosus (SLE) patients suggests a potential role of the MYSM1/miR-150/Flt3 pathway in the pathogenesis of SLE (Figure 3C) [31].

HSCs, which primarily reside in the bone marrow, possess the unique capabilities of self-renewal and differentiation into various blood and immune cells [34,35]. These cells can be activated during developmental stages or under stress conditions to maintain lifelong blood and immune cell production through hematopoiesis. A dysfunction in HSC function can result in bone marrow failure or a progression towards pre-cancerous and leukemic states [36,37]. To maintain continuous hematopoiesis, HSCs have developed unique physiological adaptations, including strictly regulated protein synthesis and energy production dependent on glycolysis and autophagy [38]. Recent studies have revealed that MYSM1 plays a crucial role in regulating HSC sensitivity to ferroptosis [39]. The research team led by Vijay G. Sankaran discovered that MYSM1 deficiency results in multiple adverse effects: decreased mRNA translation levels, reduced protein synthesis in HSCs, and the activation of various ferroptosis-related pathways, thereby triggering spontaneous ferroptosis in HSCs, ultimately leading to HSC depletion and congenital bone marrow failure syndrome. The research further revealed that restoring MYSM1 expression enhances protein synthesis rates and reduces HSC sensitivity to ferroptosis. This selective vulnerability to ferroptosis explains the loss of HSC function in the absence of MYSM1 and elucidates how physiological variations between cell populations contribute to differential ferroptosis sensitivity.

### 3.2. MYSM1’s Role in Neural Stem Cell Proliferation and Differentiation

Mysm1 deficiency not only disrupts the function of the normal hematopoietic system, but also leads to significant neurological abnormalities [6,7]. Recent studies have shown that MYSM1 is expressed in mouse NSCs, with expression levels progressively increasing with age [7]. When researchers utilized genetic mouse models to specifically ablate Mysm1 in NSCs, they observed abnormal brain development, characterized by microcephaly. Subsequent investigations revealed that Mysm1 deficiency simultaneously promotes NSC proliferation and apoptosis, ultimately depleting the stem cell pool [7]. Additionally, Mysm1-deficient NSCs show a distinct bias toward neuronal differentiation rather than astrocytic lineage commitment. Through mechanistic studies involving RNA sequencing and genome-wide CUT&Tag analysis, researchers demonstrated that MYSM1 regulates the transcription of Id4 through epigenetic modifications at its promoter region (Figure 3D). Notably, restoring Id4 expression mitigated the excessive proliferation and skewed the differentiation patterns observed in Mysm1-deficient NSCs, highlighting the essential role of MYSM1 in maintaining NSC homeostasis. These findings suggest that the MYSM1-Id4 axis represents a potential therapeutic target for regulating NSC proliferation and differentiation. Considering that the expression of MYSM1 increased with age and that Mysm1-deficient NSCs prompt neuron differentiation, suppressing the MYSM1-Id4 axis maybe an ideal strategy for stimulating NSC proliferation and neuron development.

### 3.3. MYSM1’s Role in Regulating Mesenchymal Stem Cell Proliferation, Differentiation, and Immune Modulation

MYSM1 has been shown to play a critical role in the differentiation and maintenance of HSCs and mesenchymal stem cells (MSCs). MSCs, which reside in the bone marrow, are multipotent stromal cells that function as progenitors for osteoblasts, chondrocytes, adipocytes, and myocytes [40,41,42]. Studies have demonstrated that Mysm1-deficient mice exhibit a significantly reduced bone mass in both long and cranial bones when compared to their wild-type counterparts. Furthermore, MSCs deficient in Mysm1 display abnormal differentiation and accelerated adipogenesis, emphasizing MYSM1’s essential role in MSC homeostasis and differentiation. These findings highlight its potential as a therapeutic target for treating metabolic bone diseases, such as osteoporosis [14].

Like other stem cells, MSCs rely on essential transcriptional regulators to orchestrate their proliferation and lineage commitment. Studies have demonstrated that a loss of p53 can compensate for the developmental bone defects observed in Mysm1-deficient mice [43,44]. While Mysm1-deficient mice exhibit bone deformities and osteoporosis, their MSCs maintain normal osteogenic differentiation capacities in vitro. Remarkably, the concurrent knockout of p53 fully reverses the skeletal abnormalities and osteoporosis in Mysm1-deficient mice, even though the deletion of p53 does not restore Runx2 expression in Mysm1-deficient osteoblasts.

Beyond regulating MSC proliferation and differentiation, MYSM1 also modulates MSC-mediated immune responses. Adipose-derived mesenchymal stem cells (AD-MSCs) are particularly valuable for cell-based therapies for tissue repair and regeneration due to their multilineage differentiation capacity and immunosuppressive properties. A recent study has discovered that MYSM1 regulates the immunomodulatory functions of AD-MSCs by regulating miR-150 [45]. MYSM1 expression increased in AD-MSCs when treated with inflammatory cytokines. When Mysm1 was knocked down in AD-MSCs, their immunosuppressive capacity was significantly reduced, as evidenced by the diminished inhibition of T cell proliferation, increased pro-inflammatory factor secretion, and decreased nitric oxide (NO) production in vitro [45]. In mouse models, Mysm1-deficient AD-MSCs exacerbated inflammatory bowel disease but suppressed tumor growth. Furthermore, Mysm1-dificient MSCs showed a decreased expression of miR-150, while transduction with a lentivirus overexpressing MYSM1 enhanced the miR-150 levels. This research reveals a novel role for MYSM1 in modulating the immunomodulatory activity of MSCs, potentially offering new avenues for clinical applications. Consequently, Mysm1 knockout in MSCs may enhance the cellular immune response, laying the foundation for the further application of MSCs in immune-related diseases.

## 4. MYSM1’s Role in Regulating Immune Cells

### 4.1. MYSM1’s Role in Regulating Mononuclear Phagocytes

Mysm1-deficient mice exhibit significant developmental deficiencies in myeloid cells, characterized by reduced populations of granulocytes, monocytes, macrophages, and their progenitors in both the bone marrow and the peripheral lymphoid organs [33]. Macrophages, as important components of the immune system, play crucial roles in the primary response to pathogens, the adaptive immune response, and tissue homeostasis. These cells functionally polarize into M1 and M2 subtypes in response to infection with host mediators and microorganisms [46]. M1 macrophages produce a large amount of NO by expressing inducible nitric oxide synthase (iNOS) and are critical for clearing bacterial, viral, and fungal infections [47,48,49]. Another macrophage subtype, called M2 macrophages or alternatively activated macrophages, play an essential role in the responses to parasite infection, tumor progression, angiogenesis, and tissue remodeling [50]. Notably, Mysm1-deficient macrophages display an increased production of inflammatory cytokines and type I interferons. The deubiquitinating enzyme MYSM1 is upregulated during the development of mouse bone marrow-derived macrophages. Mysm1-deficient macrophages exhibit accelerated proliferation and increased cell death rates [51]. Under LPS stimulation, these cells produce higher levels of pro-inflammatory cytokines such as IL-1β and TNFα and express increased levels of iNOS and the surface marker CD86. In in vivo tumor models, Mysm1-deficient macrophages display characteristics of M1 macrophages and suppress tumor growth, indicating MYSM1’s crucial role in macrophage survival and polarization and meaning it could potentially serve as a target for cell therapy. Furthermore, MYSM1 directly influences macrophage activation in response to inflammatory stimuli and infections. Mysm1-deficient mice, either systemically or in the myeloid lineage, exhibit an increased susceptibility to sepsis and peritonitis while showing improved viral clearance [18]. Interestingly, this function operates independently of MYSM1’s role as a chromatin transcription regulator. Instead, it relies on a transient cytoplasmic pool of MYSM1 protein generated during macrophage activation [18]. This cytoplasmic MYSM1 removes polyubiquitin chains from TRAF3, TRAF6, and RIP2, effectively suppressing the TLR and NOD2 signaling pathways and inhibiting the cGAS-STING pathway, thereby regulating innate and autoimmune responses [18,28,52].

Dendritic cells, the most crucial antigen-presenting cells in the immune system, play a critical role in activating naïve T cells. In Mysm1-deficient mice, the number of DCs in lymphoid organs is significantly reduced, while the development of granulocytes and macrophages remains largely unaffected [33]. The absence of MYSM1 results in a decrease in hematopoietic progenitors and DC precursors, leading to impaired DC differentiation induced by the Flt3 ligand [33]. Molecular studies have shown that MYSM1 maintains Flt3 expression by regulating histone modifications at the Flt3 gene promoter, thereby affecting DC development. This study reveals the key role of MYSM1 in the epigenetic regulation of Flt3 transcription and DC development, while also uncovering a novel mechanism for lineage determination from common myeloid progenitors (CMPs). MYSM1 is also essential for the development of DCs, including both conventional and plasmacytoid lineages [53]. MYSM1, acting as a chromatin-bound deubiquitinating enzyme, plays a role in the development of hematopoietic precursors in both mice and humans, controlling the development of DCs and their response to microbial stimuli. The expression and catalytic activity of MYSM1 in DCs are essential for maintaining DC numbers in vivo or for DC activation in response to microbial stimulation. Specifically, MYSM1 exerts its DUB catalytic activity in hematopoietic progenitors to enable normal DC lineage development. The absence of MYSM1 not only causes severe DC depletion but also results in the production of functionally compromised DCs. This deficiency leads to the dysregulation of numerous housekeeping transcriptional programs and significantly altered responses to microbial stimulation.

### 4.2. MYSM1’s Role in Regulating Lymphocytes

One of the key features of Mysm1 deficiency, observed in both human patients and mouse models, is the severe depletion of B cell lineages [54,55]. The Ebf1 gene, which encodes a crucial transcription factor for B cell lineage specification, is directly implicated in the early B cell development arrest in Mysm1-deficient mice, due to reduced Ebf1 expression. Notably, the retroviral expression of Ebf1 in the bone marrow of Mysm1-deficient mice partially rescues B cell development [30]. In Mysm1-deficient conditions, the depletion of Ebf1 correlates with the reduced recruitment of the transcription factor E2A, the enhanced recruitment of the PRC1 complex, and elevated levels of histone H2AK119ub at the Ebf1 locus. Additionally, the interaction between MYSM1 and the SWI/SNF chromatin remodeling complex components BRM and BRG1 is also believed to play a vital role in regulating Ebf1 expression.

Despite the substantial depletion of B cells, Mysm1-deficient mice maintain normal antibody levels and produce antigen-specific antibodies in response to immune challenges, exhibiting an increased frequency of antigen-specific plasma cells in their lymphoid organs. These Mysm1-deficient plasma cells exhibit enhanced antibody secretion levels in vitro and display altered gene expression patterns, characterized by downregulated B cell lineage transcription factors, Pax5 and Bach2, alongside an increased expression of the plasma cell transcription factors, Blimp1 and Xbp1. MYSM1 functions through histone H2AK119 deubiquitination and recruits the transcription factor PU.1 to the Pax5 locus, thereby suppressing plasma cell differentiation by maintaining Pax5 expression [3]. Additionally, MYSM1 negatively regulates B1a cell development and expansion by regulating the expression of miR-150 in coordination with the transcription factor cMYC [31]. These findings highlight MYSM1’s critical role in early B cell lineage differentiation and its minor regulatory effect on plasma cell development during B cell-mediated immune responses.

Studies utilizing Mysm1^fl/fl^Tg.mb1-cre mice demonstrated that the deletion of Mysm1 at the pro-B cell stage resulted in an approximately twofold reduction in B cell numbers within lymphoid organs, accompanied by an increased expression of activation markers, impaired survival and proliferation, and altered gene expression profiles [55]. In later stages, the absence of MYSM1 had no significant effect on B cell numbers or responses to stimulation. Therefore, MYSM1 is essential for B cell lineage specification but dispensable in the later stages of development. Importantly, MYSM1 activity at the prepro-B cell stage of development is important for the normal programming of B cell responses to stimulation once they complete their maturation process.

Additionally, several studies have investigated T cell and NK cell differentiation defects in Mysm1-deficient mouse models. Mysm1-deficient mice exhibit a marked reduction in mature NK cells within the bone marrow, blood, and lymphoid organs, a defect attributed to the intrinsic role of MYSM1 in inducing the expression of the transcription factor Id2; its deficiency correlates with the diminished recruitment of the transcription factor NFIL3, an increased association with the PRC1 complex, and elevated levels of H2AK119ub [5]. In Mysm1-deficient mice, the absolute number of T cells in the peripheral lymphoid organs is significantly reduced, which is associated with a severe reduction in the cellularity of the thymus and a depletion of all thymocyte subsets [4,13,56], including the early thymic progenitors (ETPs) [4]. The potential cellular mechanisms may include the upstream defects in lymphoid lineage specification within the bone marrow [4,13,56], as well as increased levels of cell apoptosis within the thymus [4]. At the molecular level, activation of the p53-stress response was extensively characterized and shown to be functionally important, based on the rescue of T cell development in Mysm1-/-p53-/- mice [4,57]. The suggested triggers for p53 activation in Mysm1-deficient mice include the putative roles of MYSM1 in the maintenance of p19ARF expression within the thymus [4] and IRF2 and IRF8 expression in bone marrow progenitor cells [56].

Research has discovered that the histone H2A deubiquitinating enzyme MYSM1 plays a crucial role in the maintenance, activation, and survival of CD8^+^ T cells. In a mouse model with conditional deletion of Mysm1 it was observed that the number of CD8^+^ T cells was reduced by half, and the cells exhibited a hyperactivated state with impaired proliferation, increased production of pro-inflammatory cytokines, and elevated apoptosis, along with upregulation of the p53 tumor suppressor protein, suggesting that the mechanisms for CD8^+^ T cell dysfunction in this model might also be p53-dependent. In an experimental cerebral malaria model, mice with an Mysm1 deficiency had a sustained decrease in CD8^+^ T cell numbers after infection, but showed improved survival rates after a parasitic challenge. These results indicate that MYSM1 is a novel factor in the immune system for CD8^+^ T cells, enhancing our understanding of the role of histone H2A deubiquitinating enzymes in the biology of cytotoxic T cells.

## 5. MYSM1’s Role in Regulating Various Diseases

### 5.1. MYSM1 Mutation and Inherited Bone Marrow Failure Syndrome

**Figure 4:**
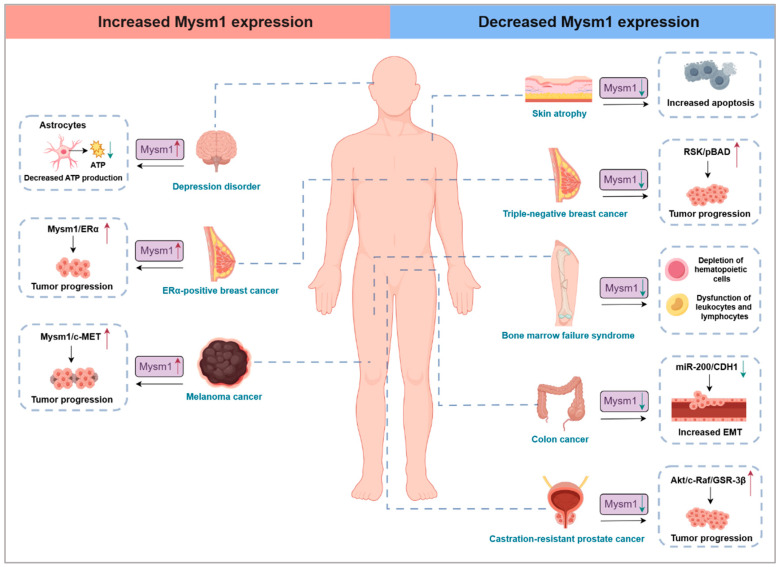
MYSM1’s critical roles in regulating the development and function of various tissues and organs.

MYSM1 is involved in regulating various types of diseases, such as inherited bone marrow failure syndrome (IBMFS), cancer, aging, and depression (Figure 4). Firstly, an Mysm1 deficiency has been associated with IBMFS [58]. A study that characterized five patients with homozygous Mysm1 mutations revealed that all patients exhibited anemia and leukopenia, with some also presenting with growth retardation, developmental abnormalities, and neurological delays [59,60,61]. A severe depletion of B cells was observed in all five patients, and most displayed reduced NK cell populations, with three experiencing neutropenia and two showing T cell exhaustion. Additionally, skeletal and craniofacial abnormalities, such as limb shortening and midfacial hypoplasia, were noted in two patients. Mysm1-deficient mice exhibit partial embryonic lethality, growth retardation, and skeletal and coat color pigmentation defects, alongside complex hematopoietic and immune phenotypes. In contrast, heterozygous Mysm1 mice exhibit no phenotypic abnormalities [62,63,64]. The high sequence homology between humans and mice renders Mysm1-deficient mouse models invaluable for uncovering the fundamental physiological roles of MYSM1 in mammals.

An Mysm1 deficiency in these mice leads to elevated levels of the p53 protein in multiple hematopoietic cell types, thereby impairing HSC function and lymphopoiesis. In double-knockout Mysm1-/-p53-/- mice, p53 deletion completely rescues the developmental and hematopoietic defects attributed to Mysm1 deficiency, including the recovery of lymphopoiesis and both the numbers and function of HSCs [65]. This suggests that p53 activation is the predominant mechanism driving the hematopoietic abnormalities associated with an Mysm1 deficiency.

### 5.2. MYSM1’s Role in Cancer Development and Progression

Previous studies have explored the role of MYSM1 in various cancer models, revealing its potential oncogenic and tumor-suppressive properties (Table 1). Studies indicate that a deficiency or reduced expression of MYSM1 in several cancers suggest its tumor-suppressive function. Notably, Mysm1 deficiency activates p53 in hematopoietic cells, implying that MYSM1 typically suppresses p53 activation and could be targeted for therapy in hematological malignancies with active p53 or intact wild-type p53 function [4,57]. Mysm1-deficient mice develop spontaneous thymic lymphomas at 6–9 months of age; however, these tumors have not been thoroughly characterized and may contain genetic changes that disrupt the p53 stress response pathway [66]. In colorectal cancer, a lower MYSM1 expression is observed, and its high expression correlates with a favorable prognosis [9]. Mechanistic studies indicate that MYSM1 may epigenetically enhance the expression of the miR-200 family and CDH1, thus inhibiting the PI3K/AKT signaling pathway and reducing the progression of colorectal cancer. Additionally, MYSM1 is downregulated in CRPC, where its decreased expression under androgen ablation conditions facilitates the proliferation of CRPC cells and impedes cellular senescence [8]. Furthermore, MYSM1 expression is significantly reduced in TNBC, and restoring MYSM1 expression increases cisplatin-induced apoptosis in these cells [12].

Conversely, several studies have demonstrated that elevated MYSM1 expression is associated with cancer progression, enhancing tumor growth. For instance, elevated levels of the MYSM1 protein have been observed in human melanomas compared to normal melanocytes, and the knockdown of Mysm1 impairs the proliferation and survival of melanoma cell lines [10]. Additionally, an increased MYSM1 expression has been observed in estrogen receptor-alpha (ERα)-positive breast cancer (BCa) clinical samples [11]. Mysm1 deficiency attenuates the growth of BCa-derived cells in xenograft models and increases the sensitivity of BCa cells to anti-estrogen drugs. Although the mechanisms underlying MYSM1’s role in tumorigenesis remain limited, these studies suggest that MYSM1 expression is crucial for assessing a clinical prognosis and may serve as a prognostic biomarker and therapeutic target for various cancers. However, the limited sample size and young age of the patients suggest that our comprehension of the cancer susceptibility associated with Mysm1 deficiency in humans may be incomplete.

### 5.3. MYSM1 as a Key Inhibitor of Aging

The cellular and molecular mechanisms of aging are pivotal in preventing age-related decline [67,68]. Research has shown that MYSM1 is an important inhibitor of aging and age-related pathologies. MYSM1 significantly suppresses cellular senescence, delays the onset of age-related diseases, and slows the aging process, thereby prolonging lifespan [69]. The ability of MYSM1 to inhibit senescence in primary human and mouse cells, along with the fact that it delays the pathological progression of tissues and organs in mice, suggest that MYSM1 is a promising anti-aging factor. It shows potential as an innovative therapeutic agent for preventing age-related diseases and extending a healthy lifespan. However, it remains unclear whether the effects of MYSM1 on aging are due to dysfunctions in tissue and organ systems stemming from its deficiency, or whether MYSM1 directly regulates aging, warranting further investigation.

### 5.4. Preliminary Exploration of MYSM1 in the Nervous System

Human patients with an Mysm1 deficiency exhibit delayed neurological development, highlighting MYSM1’s critical role in the nervous system. Research employing depression mouse models indicates that MYSM1 is highly expressed in the medial habenula (MHB) of depressed mice [6]. Knocking down MYSM1 in the brains of these mice alleviates depressive-like behaviors. Notably, most MYSM1 protein colocalizes with the astrocyte marker GFAP. The targeted knockdown of Mysm1 in the astrocytes of depressed mice enhances ATP production and activates phosphorylated p53 and AMPK, thereby ameliorating depressive symptoms. This study underscores the vital function of MYSM1 in the nervous system and emphasizes astrocytic MYSM1 as a potential risk factor for depression and a promising therapeutic target. Additionally, increased MYSM1 expression has been observed in mouse models of chronic restraint stress (CRS), Alzheimer’s disease (AD), and Parkinson’s disease (PD) (unpublished data). However, whether MYSM1 exhibits similar regulatory functions in these models as in depressive mice requires further investigation.

## 6. Conclusions and Future Directions

MYSM1 plays a pivotal role in regulating gene transcription and post-translational modifications, which are essential for tissue development and disease progression. Future research on MYSM1 should address several critical areas. Firstly, regarding regulatory mechanisms, it is vital to explore the additional ubiquitination modifications governed by MYSM1 and identify its molecular collaborators beyond its established role in removing the monoubiquitination of histone H2A at K119 and polyubiquitin chains at K63 and K27. Secondly, given MYSM1’s involvement in hematopoiesis, immune regulation, neural development, and tumor development, further investigation is needed to elucidate the specific tissues and organs directly influenced by MYSM1 in terms of development and function. Thirdly, since abnormal MYSM1 expression leads to cellular dysfunction, future studies should explore the conditions that trigger the downregulation of MYSM1 expression and the stressors that lead to its abnormal elevation. Fourthly, research should identify the molecules involved in regulating MYSM1 expression and modification to understand the underlying regulatory mechanisms. Fifthly, future research should focus on developing MYSM1-targeting small molecular inhibitors and exploring its potential as a therapeutic target for various diseases.

The high sequence homology of MYSM1 across species and the consistent phenotypes observed in its deficiency highlight the evolutionary conservation of MYSM1’s essential functions. Consequently, elucidating the functional alterations of MYSM1 in small animal models may provide valuable insights into both the diagnosis and treatment of human syndromes associated with abnormal MYSM1 expression.

## Figures and Tables

**Table 1:** List of MYSM1 related cancer research.

Cancer Type (Sorts)	MYSM1 Level	Function	Highlight (Topic)	Reference
Triple-negative breast cancer (TNBC)	Low expression	Inhibit TNBC	MYSM1 is a potential target for regulating cell apoptosis and suppressing the resistance to cisplatin in TNBC.	[12]
Castration-resistant prostate cancer (CRPC)	Low expression	Inhibit CRPC	MYSM1-AR complex-mediated repression of Akt/c-Raf/GSK-3β signaling impedes castration-resistant prostate cancer growth.	[8]
Colorectal cancer (CRC)	Low expression	Inhibit CRC	MYSM1 inhibits human colorectal cancer tumorigenesis by activating miR-200 family members/CDH1 and blocking PI3K/AKT signaling.	[9]
ERα-positive breast cancer (BC)	High expression	Induce ERα-BC	MYSM1 enhances ERα action via histone and non-histone deubiquitination to promote cell proliferation and antiestrogen insensitivity in breast cancer progression.	[11]
Melanoma	High expression	Induce melanoma	MYSM1/2A-DUB is an epigenetic regulator in human melanoma and contributes to tumor cell growth.	[10]

## Data Availability

Not applicable.

## Abbreviations

MYSM1: myb-like, SWIRM, and MPN domain 1; DUB, deubiquitinating enzyme; H2AK119ub: histone H2A monoubiquitination at lysine 119; PRC1: polycomb repression complex 1; TLR: Toll-like receptors; HSCs: hematopoietic stem cells; HPCs: hematopoietic progenitor Cells; NSCs: neural stem cells; MSCs: mesenchymal stem cells; CMPs: common myeloid progenitors; DCs: dendritic cells; ETPs: early thymic progenitors; IBMFS: inherited bone marrow failure syndrome; CRPC: castration-resistant prostate cancer; TNBC: triple-negative breast cancer; ERα-BCa: estrogen receptor-alpha-positive breast cancer; MHB: medial habenula; CRS: chronic restraint stress; AD: Alzheimer’s disease; PD: Parkinson’s disease.
